# Cisplatin neurotoxicity in the treatment of metastatic germ cell tumour: time course and prognosis

**DOI:** 10.1054/bjoc.2001.2006

**Published:** 2001-09

**Authors:** M von Schlippe, C J Fowler, S J Harland

**Affiliations:** Department of Oncology, University College, London, WIN 8AA; Institute of Urology and Nephrology, University College, London, WIN 8AA, UK

## Abstract

In order to ascertain the incidence and prognosis of cisplatin-induced neurotoxicity in testis cancer patients undergoing combination chemotherapy, 29 patients with metastatic disease were studied prospectively. Assessments included enquiry into neurological symptoms, measurement of sural nerve sensory action potential and conduction velocity, and vibration threshold in the left big toe. At the end of chemotherapy (3 to 4 cycles) only 3 out of 26 (11%) patients had paraesthesiae, but 3 months later the proportion rose to 65%. Resolution occurred in the majority over the ensuing 12 months so that only 17% had persistent symptoms. None of the 11 patients treated with 3 cycles of chemotherapy had persisting symptoms. Vibration thresholds showed a significant deterioration during chemotherapy (*P* = 0.032), further deterioration in the 3 months following chemotherapy (*P* = 0.009) and significant improvement between 3 and 12 months after chemotherapy (*P* = 0.038). Sural nerve sensory action potentials and conduction velocities were unhelpful.© 2001 Cancer Research Campaignhttp://www.bjcancer.com


British Journal of Cancer (2001) 85(6), 823–826
© 2001 Cancer Research Campaign
doi: 10.1054/ bjoc.2001.2006, available online at http://www.idealibrary.com on IDEAL
http://www.bjcancer.com

### Cisplatin neurotoxicity in the treatment of metastatic germ cell tumour: time course and prognosis

**M von Schlippe[1], CJ Fowler[2] and SJ Harland[1,2]**

[1]Department of Oncology, University College, London, WIN 8AA; [2]Institute of Urology and Nephrology, University College, London, WIN 8AA, UK

**Summary** In order to ascertain the incidence and prognosis of cisplatin-induced neurotoxicity in testis cancer patients undergoing combination chemotherapy, 29 patients with metastatic disease were studied prospectively. Assessments included enquiry into neurological symptoms, measurement of sural nerve sensory action potential and conduction velocity, and vibration threshold in the left big toe. At the end of chemotherapy (3 to 4 cycles) only 3 out of 26 (11%) patients had paraesthesiae, but 3 months later the proportion rose to 65%. Resolution occurred in the majority over the ensuing 12 months so that only 17% had persistent symptoms. None of the 11 patients treated with 3 cycles of chemotherapy had persisting symptoms. Vibration thresholds showed a significant deterioration during chemotherapy ($P$ = 0.032), further deterioration in the 3 months following chemotherapy ($P$ = 0.009) and significant improvement between 3 and 12 months after chemotherapy ($P$ = 0.038). Sural nerve sensory action potentials and conduction velocities were unhelpful. ©2001 Cancer Research Campaign http://www.bjcancer.com

Testicular cancer is the most common cancer in men between the ages of 15 and 35. Before the development of platinum-based chemotherapy in the 1970s and 1980s (Einhorn and Donohue, 1977; Einhorn and Williams, 1980), metastatic germ cell tumours of the testis were almost universally fatal. Now that the majority of these cases are cured, consideration of the toxicity of treatment takes on a greater importance.

Cisplatin, an essential component of chemotherapy for metastatic germ cell tumours, is a toxic drug. Initially, the dominant toxicities were nephrotoxicity and vomiting, but these are now usually well controlled with saline diuresis (Hardaker et al, 1974) and potent antiemetics. Neurotoxicity is also common (Kedar et al, 1978) and can be a significant cause of morbidity. Though usually mild it can occasionally be dose limiting. More commonly however the symptoms of neurotoxicity appear shortly after the end of treatment and persist for several months. There are few data on this in patients with germ cell tumour and none on the time course of the symptoms. It can therefore be difficult to advise patients on the chances of developing neurotoxicity and on its prognosis once it has developed.

Neurotoxicity is usually assessed in patients receiving chemotherapy by enquiring about symptoms and by physical examination. The most sensitive indicator of cisplatin-induced peripheral neuropathy is the vibration sensation threshold, which may be elevated even in asymptomatic patients (Ashraf et al, 1983). Nerve conduction studies, looking at both conduction velocities and action potentials, are also commonly performed (e.g. Cavaletti et al, 1992). We therefore performed a prospective longitudinal study on men undergoing chemotherapy for metastatic germ cell tumour to determine the incidence, time course and reversibility of neurotoxicity.

*Received 28 February 2001*
*Revised 21 June 2001*
*Accepted 5 July 2001*

*Correspondence to:* SJ Harland

### METHODS

### Patients

29 patients with testicular germ cell tumour requiring cytotoxic chemotherapy (median age 30 years, range 14–50 years) were studied. Most patients received 3 or 4 cycles of BEP (bleomycin, etoposide and cisplatin), each cycle containing cisplatin 100 mg $m^{-2}$ (Einhorn et al, 1987). Patients with seminoma received chemotherapy with less bleomycin. 2 patients were treated with BOP-VIP (bleomycin, vincristine and cisplatin alternating with etoposide, ifosfamide and cisplatin) (Kaye et al, 1998), and 2 received lower doses of cisplatin than intended (not because of neurotoxicity). One patient received high-dose chemotherapy after initial treatment.

### Clinical assessment

Careful enquiry was made concerning the presence of numbness and paraesthesiae during the follow-up period, initially at 6-week intervals extending to 2 monthly after 6 months. 27 sets of case notes were examined for these. The median follow-up was 4 years (range 2–8 years).

### Neurophysiology

Vibration sensation threshold was determined using the Biothesiometer (Bio-Medical, Ohio). A cylindrical stylus, 13 mm in diameter, vibrating at 100 Hz, was allowed to rest on the patient's great toe. The amplitude of vibration was varied by the operator, and the patient was asked to report the appearance or disappearance of the vibration (Nielsen, 1972). The readings were read off the dial and expressed in 'biothesiometer units'. This technique has good reproducibility, suitable for serial measurements in clinical trials (Le Quesne and Fowler, 1987). Biothesiometry was performed at the beginning and at the end of chemotherapy, and at intervals subsequently. By 12 months following the end of chemotherapy, 29 patients had undergone 81 tests. The sural nerve

**Table 1** Occurrence and resolution of neurological symptoms according to total cisplatin (CDDP) dose

| Cumulative cisplatin dose (mg m$^{-2}$) | Symptoms | | | | |
|---|---|---|---|---|---|
| | None | Resolved | Persisted | No data | Total |
| <300 | 0 | 2 | 0 | 1 | 3 |
| 300 | 7 | 4 | 0 | 0 | 11 |
| 400 and above | 2 | 6 | 5* | 2 | 15 |
| Total | 9 | 12 | 5 | 3 | 29 |

* 2 patients also received a total dose of vincristine 6 mg m$^{-2}$.
This table with only minor variations was previously published elsewhere (von Schlippe et al, 1998).

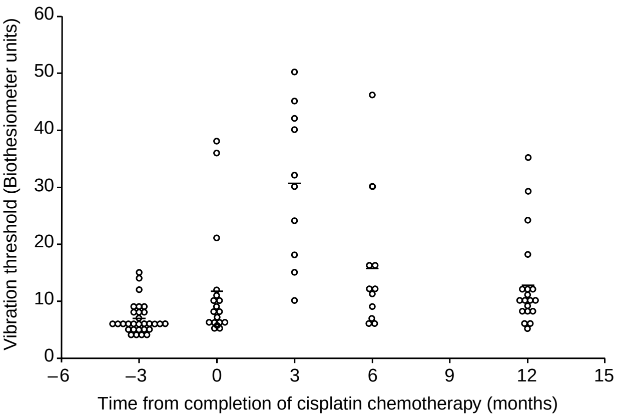

**Figure 2** Variation of vibration threshold in the left big toe with cisplatin chemotherapy. Symbols: o vibration threshold; — mean vibration threshold (in Biothesiometer units)

sensory action potential and conduction velocity were measured using surface electrodes (di Benedetto, 1970). Test results were grouped according to the nearest 3 month time period. Statistical evaluation of the mean changes in value from baseline was performed using a paired *t*-test.

### RESULTS

Data on symptoms were available on 26 of the 29 patients studied (Table 1, Figure 1). Review of patient records showed that 3 patients had paraesthesiae at the end of treatment. A further 14 became symptomatic later (3 at 1 month, 1 at 2 months, and the remaining 10 at 3 months). All neurotoxicity was WHO Grade I (mild paraesthesiae) apart from 2 cases which were Grade II (severe paraesthesiae). Resolution of symptoms was documented a mean of 4 months later (range 2 weeks to 1 year after the onset of symptoms) in 12 patients.

5 patients had persistent symptoms. 2 had received vincristine as part of the BOP-VIP regimen, and 1 had received high-dose chemotherapy with carboplatin, etoposide and cyclophosphamide on relapse. 2 others had received a cumulative dose of cisplatin of 400 and 600 mg m$^{-2}$.

The time course of reported symptoms correlated well with changes in the vibration threshold (Figure 2). By the end of treatment, a modest rise in vibration threshold could be seen. The vibration threshold peaked 3 months later, and then began to resolve, with a substantial improvement at 6 months, and almost complete recovery at 18 months. Statistical analysis of the paired data out to 12 months after the completion of chemotherapy (Table 2) confirmed that vibration threshold was higher than before the start of chemotherapy. The vibration threshold at 3 and 6 months was significantly worse than immediately after the end of chemotherapy. By 12 months, however, significant improvement in the vibration threshold had occurred as compared to the vibration threshold at 3 months (Table 2).

There was an apparent relationship between the severity of neurotoxicity and the total cisplatin dose (Figure 3). However, small numbers prevented statistical confirmation of this observation.

We also examined the changes in sural nerve sensory action potential (Figure 4A) and conduction velocity (Figure 4B). No significant deviations from baseline could be detected in either case.

### DISCUSSION

The neurotoxicity of cisplatin poses a significant clinical problem. Symptoms may be troublesome and persistent, and may even lead to treatment with cisplatin being curtailed. Together with nephrotoxicity, neurotoxicity also limits the scope for dose intensification

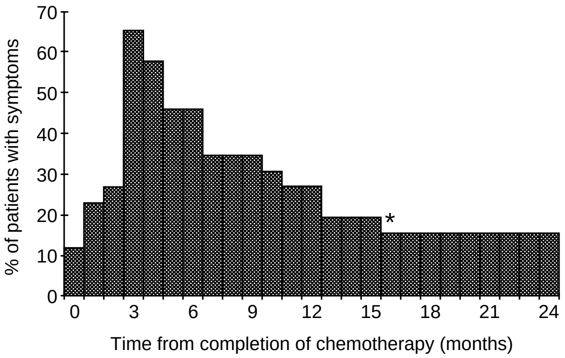

**Figure 1** Proportion of patients with neurological symptoms after completion of chemotherapy. *Patient received high dose chemotherapy at 12 months, therefore censored at 12 months

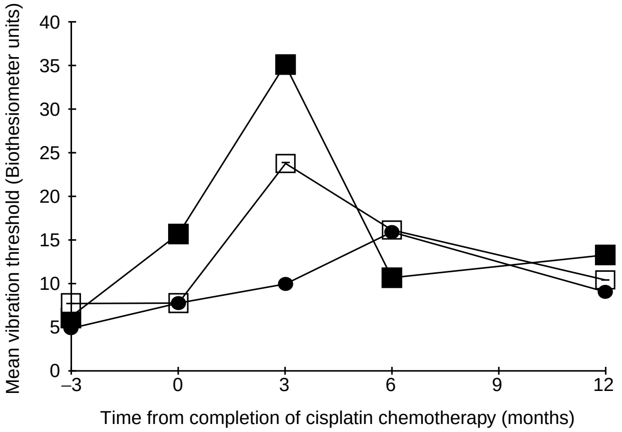

**Figure 3** Dependence of the variation in vibration threshold in the left big toe on the cumulative dose of cisplatin. Symbols: ● cisplatin <300 mg m$^{-2}$; ❒ cisplatin 300 mg m$^{-2}$; ■ cisplatin >300 mg m$^{-2}$

© 2001 Cancer Research Campaign

**Table 2** Vibration threshold of right big toe in relation to time of chemotherapy: paired data

| Time points | Mean difference ± SE in biothesiometry units | Paired observations | *P* value |
|---|---|---|---|
| Pre-chemo vs end-chemo | 6.3 ± 2.6 | 16 | 0.032 |
| Pre-chemo vs 3 months post- | 19.0 ± 3.7 | 11 | < 0.001 |
| Pre-chemo vs 6 months post- | 7.1 ± 1.7 | 9 | 0.003 |
| Pre-chemo vs 12 months post- | 5.4 ± 1.8 | 16 | 0.009 |
| Pre-chemo vs 18 months post- | 2.3 ± 1.2 | 3 | NS |
| End-chemo vs 3 months post- | 11.8 ± 2.9 | 6 | 0.009 |
| End-chemo vs 6 months post- | 5.2 ± 1.7 | 5 | 0.035 |
| 3 months post- vs 12 months post- | –8.0 ± 3.0 | 7 | 0.038 |

with cisplatin. In our study, we found that 5 out of 27 patients on whom data were available remained symptomatic, an average of 5 years after the end of chemotherapy. Bokemeyer et al found that 17% of patients treated for testicular cancer who had been in complete remission for at least 1 year still had symptoms of neuropathy, 5% grading them as severe (Bokemeyer et al, 1996). We also found that 12 of the 27 patients developed symptoms only after the end of chemotherapy. Others have also found that the symptoms of cisplatin neuropathy can progress after the end of chemotherapy (Mollman et al, 1988; Grunberg et al, 1989), with variable and unpredictable degrees of recovery.

Because patients with metastatic germ cell tumour tend to receive only 3 or 4 cycles of chemotherapy, they offer an opportunity to study the development of cisplatin-induced neuropathy

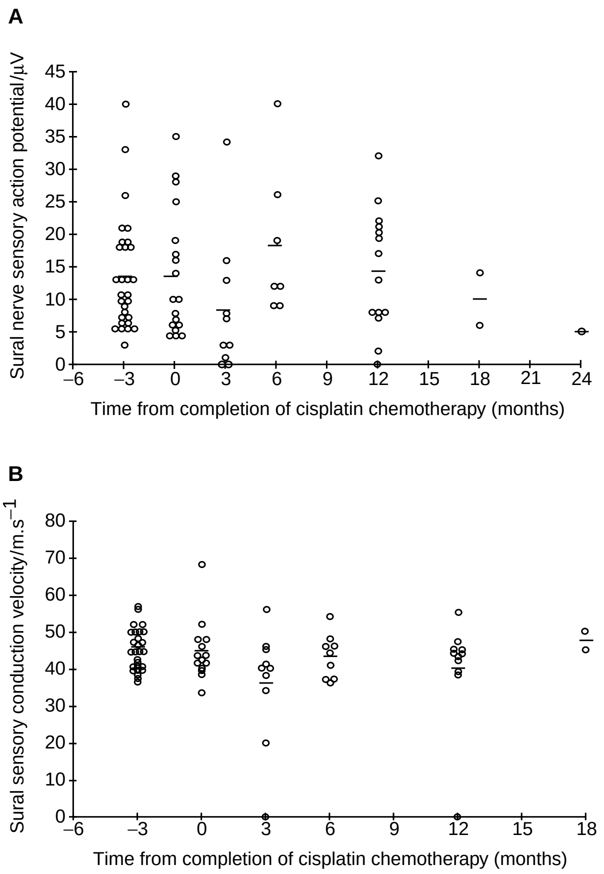

**Figure 4** Sural nerve neurophysiology: variations in sensory action potential **(A)** and nerve conduction velocity **(B)** after completion of chemotherapy. Symbols: o sural nerve sensory action potential **(A)** or nerve conduction velocity **(B)**; — mean values even after chemotherapy has ended. We were able to correlate the development of symptomatic neuropathy with a deterioration in biothesiometry, and to follow their concurrent recovery. The Biothesiometer, introduced into clinical practice in the 1950s (Steiness, 1957a, 1957b), has been found to be as reliable as other instruments in the assessment of vibration sensation (Le Quesne and Fowler, 1987). Daugaard et al used the Biothesiometer in 8 patients undergoing BEP chemotherapy for germ cell tumours. Abnormal biothesiometry was found in all the patients at the end of 3 cycles of chemotherapy, and 4 patients had symptoms of neuropathy (Daugaard et al, 1987). However, the biothesiometry in these patients was not followed after the end of chemotherapy.

In this study, we found that our patients' symptoms followed the changes in biothesiometry well. By contrast, neither the sural nerve sensory action potential or sural nerve conduction velocity correlated with either patients' symptoms or their biothesiometry. These methods have, however, been used frequently by other investigators in the study of cisplatin neuropathy (e.g. Riggs et al, 1988; Cavaletti et al, 1994). They have shown decreases in the sensory nerve action potential amplitude, although with wide variation. Motor parameters appear to be affected much less, and this is borne out by the clinical observation that weakness due to motoneurone damage is a late event that is rarely seen in response to cisplatin chemotherapy.

In this study, all patients receiving a cumulative dose of 300 mg $m^{-2}$ cisplatin had a full resolution of any neuropathy, and only 2 out of 11 patients receiving a cumulative dose of 400 mg $m^{-2}$ had persistent symptoms (Table 1). We have reported previously that neurotoxicity appears to correlate with cumulative cisplatin dose (von Schlippe et al, 1998). Certainly when 4 cycles of BEP chemotherapy are compared with 3 in large numbers of patients, the incidence of sensory symptoms is significantly higher (de Wit et al, 2001). The role of dose intensity has been studied (Cavaletti et al, 1992; Hilkens et al, 1995), but its significance is still unresolved.

Our results illustrate the damaging effects of concurrent treatment with other neurotoxic drugs, as 2 of the 3 patients treated with vincristine developed persistent symptomatic neurotoxicity. The advent of the taxanes, which have both a wide spectrum of activity and significant neurotoxic side-effects, may lead to an increase in neurotoxicity (Berger et al, 1997; Hilkens et al, 1997). Neuroprotective agents are being investigated, including amifostine (Foster-Nora and Siden, 1997; Planting et al, 1999) and the ACTH analogue Org 2766 (Gerritsen van der Hoop et al, 1990). As yet, none of these compounds is used routinely, and they can have significant side-effects. Biothesiometry can provide a convenient measure of their efficacy.

Our results suggest that, if cisplatin is the only neurotoxic drug in the regimen, full recovery of neurotoxicity is likely up to a

© 2001 Cancer Research Campaign cumulative dose of 400 mg m$^{-2}$ of cisplatin without the use of neuroprotective agents. This is likely to apply to the majority of patients with metastatic germ cell tumour, most of whom will be cured.

In the event of disease relapse there should be a greater concern about the development of long-term paraesthesiae. Whereas biothesiometry will be able to provide an objective index of neurotoxicity it may not be of value in management since there is a overriding need to give aggressive treatments despite non-lethal toxicity and because the full extent of nerve damage is unlikely to be detected until 2–4 months after the completion of treatment.

### ACKNOWLEDGEMENTS

We would like to thank Sister Liz Fenwick for co-ordinating studies, Mary Boland for performing the biothesiometry measurements, Dr Paul Nicholson for carrying out the statistical analysis and Rose Clark for typing the manuscript.

### REFERENCES

Ashraf M, Scotchel PL, Krall JM and Flink EB (1983) Cis-platinum-induced hypomagnesemia and peripheral neuropathy. *Gynecol Oncol* **16**: 309–318

Berger T, Malayeri R, Doppelbauer A, Krajnik G, Huber H, Auff E and Pirker R (1997) Neurological monitoring of neurotoxicity induced by paclitaxel/cisplatin chemotherapy. *Euro J Cancer* **33**: 1393–1399

Bokemeyer C, Berger CC, Kuczyk MA and Schmoll HJ (1996) Evaluation of long-term toxicity after chemotherapy for testicular cancer. *J Clin Oncol* **14**: 2923–2932

Cavaletti G, Marzorati L, Bogliun G, Colombo N, Marzola M, Pittelli MR and Tredici G (1992) Cisplatin-induced peripheral neurotoxicity is dependent on total-dose intensity and single-dose intensity. *Cancer* **69**: 203–207

Cavaletti G, Bogliun G, Marzorati L, Tredici G, Colombo N, Parma G and Miceli MD (1994) Long-term peripheral neurotoxicity of cisplatin in patients with succesfully treated epithelial ovarian cancer. *Anticancer Res* **14**: 1287–1292

Daugaard GK, Petrera J and Trojaborg W (1987) Electrophysiological study of the peripheral and central neurotoxic effect of cis-platin. *Acta Neurol Scand* **76**: 86–93

de Wit R, Roberts JT, Wilkinson PM, de Mulder PHM, Mead GM, Fossa SD, Cook P, de Prijck L, Stenning S and Collete L (2001) Equivalence of three or four cycles of bleomycin, etoposide and cisplatin chemotherapy and of a 3-or 5-day schedule in good-prognosis germ cell cancer: a randomised study of the European Organisation for Research and Treatment of Cancer Genitourinary Tract Cancer Co-operative Group and the Medical Research Council. *J Clin Oncol* **19**: 1629–1640

di Benedetto M (1970) Sensory nerve conduction in lower extremeties. *Arch Phys Med* **51**: 253–258

Einhorn LH and Donohue J (1977) Cis-diammine-dichloroplatinum, vinblastine, and bleomycin combination chemotherapy in disseminated testicular cancer. *Ann Intern Med* **87**: 293–298

Einhorn LH and Williams SD (1980) Chemotherapy of disseminated testicular cancer: a random prospective study. *Cancer* **46**: 1339–1344

Einhorn LH, Williams SD, Loehrer PJ, Birch R, Drasga R, Omuna G and Greco FA (1989) Evaluation of optimal duration of chemotherapy in favourable-prognostic disseminated germ cell tumours: bleomycin and either vinblastine or etoposide. *J Clin Oncol* **7**: 387–391

Foster-Nora JA and Siden R (1997) Amifostine for protection from antineoplastic drug toxicity. *Am J Health-System Pharm* **54**: 787–800

Gerritsen van der Hoop R, Vecht CJ, van der Burg MEL, Elderson A, Boogerd W, Heimans JJ, Vries EP, van Houwelingen JC, Jennekens FGI, Gispen WH and Neijt JP (1990) Prevention of cisplatin neurotoxicity with an ACTH(4–9) analogue in patients with ovarian cancer. *New Engl J Med* **322**: 89–94

Grunberg SM, Sonka S, Stevenson LL and Muggia FM (1989) Progressive paresthesias after cessation of therapy with very high-dose cisplatin. *Cancer Chemo Pharmacol* **25**: 62–64

Hardaker WT, Stone RA and McCoy R (1974) Platinum nephrotoxicity. *Cancer* **34**: 1030–1032

Hilkens PHE, van der Burg MEL, Moll JWB, Planting AST, van Putten WLJ, Vecht CJ and van den Bent MJ (1995) Neurotoxicity is not enhanced by increased dose intensities of cisplatin administration. *Euro J Cancer* **31A**: 678–681

Hilkens PH, Pronk LC, Verweij J, Vecht CJ, van Putten WL and van den Bent MJ (1997) Peripheral neuropathy induced by combination chemotherapy of docetaxel and cisplatin. *Brit J Cancer* **75**: 417–22

Kaye SB, Mead GM, Fossa S, Cullen M, de Wit R, Bodrogi I, van Groeningen C, Sylvester R, Collette L, Stenning S, De Prijck L, Lallemand E and de Mulder P (1998) Intensive induction-sequential chemotherapy with BOP/VIP-B compared with treatment with BEP/EP for poor-prognosis metastatic nonseminomatous germ cell tumour: a randomised Medical Research Council/European Organisation for Research and Treatment of Cancer study. *J Clin Oncol* **16**: 692–701

Kedar A, Cohen ME and Freeman AI (1978) Peripheral neuropathy as a complication of cis-dichlorodiammineplatinum(II) treatment: a case report. *Cancer Treat Rep* **62**: 819–821

Le Quesne PM and Fowler CJ (1987) Quantitative evaluation of toxic neuropathies in man. In: Ellingson RJ, Murray N, Halliday M, eds. The London Symposium. *EEG J Suppl* **39**: 347–354

Mollman JE, Hogan WM, Glover DJ and McCluskey LF (1988) Unusual presentation of cis-platinum neuropathy. *Neurology* **38**: 488–490

Nielsen VK (1972) The peripheral nerve function in chronic renal failure. IV. An analysis of the vibratory perception threshold. *Acta Med Scand* **191**: 287–296

Planting AS, Catimel G, de Mulder PH, de Graeff A, Hoeppener F, Verweij J, Oster W and Vermorken JB (1999) Randomized study of a short course of weekly cisplatin with or without amifostine in advanced head and neck cancer. EORTC Head and Neck Cooperative Group. *Annal Oncol* **10**: 693–700

Riggs JE, Ashraf M, Snyder RD and Gutmann L (1988) Prospective nerve conduction studies in cisplatin therapy. *Annal Neurol* **23**: 92–94

Steiness IB (1957a) Vibratory perception in normal subjects. A biothesiometric study. *Acta Med Scand* **158**: 315–325

Steiness IB (1957b) Vibratory perception in diabetics. A biothesiometric study. *Acta Med Scand* **158**: 327–335

von Schlippe M, Fowler CJ and Harland SJ (1998) Cisplatin Neurotoxicity. In: Jones WG, Appleyard I, Harnden P, Joffe JK eds. Germ Cell Tumours IV. John Libbey & Co. Ltd.

*© 2001 Cancer Research Campaign*
